# Variation in ultraviolet radiation and diabetes: evidence of an epigenetic effect that modulates diabetics’ lifespan

**DOI:** 10.1186/1868-7083-5-5

**Published:** 2013-04-02

**Authors:** George E Davis, Walter E Lowell

**Affiliations:** 1 Psybernetics Research Group, 28 Eastern Ave., Augusta, ME 04330, USA

**Keywords:** solar cycles, seasonality, type 2 diabetes, epigenome, UVR, ultraviolet radiation, lifespan, month of conception

## Abstract

**Background:**

Published research has shown that month-of-birth variations modulate the incidence of adult human diseases. This article explores diabetes type 2 as one of those diseases. This study uses the death records of approximately 829,000 diabetics (approximately 90% were type-2) born before the year 1945 (and dying between 1979 and 2005) to show that variations in adult lifespan vary with ultraviolet radiation (UVR) at solar cycle peaks (MAX, approximately a three-year period) with less at non-peaks (MIN, approximately an eight-year period). The MAX minus MIN (in years) was our measure of sensitivity (for example, responsiveness) to long-term variations in UVR.

**Results:**

Diabetics were less sensitive than non-diabetics, and ethnic minorities were more sensitive than whites. Diabetic males gained 6.1 years, and females 2.3 years over non-diabetics, with diabetic males gaining an average of 3.8 years over diabetic females. Most variation in lifespan occurred in those conceived around the seasonal equinoxes, suggesting that the human epigenome at conception is especially influenced by rapid variation in UVR. With rapidly decreasing UVR at conception, lifespan decreased in the better-nourished, white, female diabetic population.

**Conclusions:**

Rapidly changing UVR at the equinoxes modulates the expression of an epigenome involving the conservation of energy, a mechanism especially canalized in women. Decreasing UVR at conception and early gestation stimulates energy conservation in persons we consider ‘diabetic’ in today’s environment of caloric surfeit. In the late 19th and early 20th centuries ethnic minorities had poorer nutrition, laborious work, and leaner bodies, and in that environment a calorie-conserving epigenome was a survival advantage. Ethnic minorities with a similar epigenome lived long enough to express diabetes as we define it today and exceeded the lifespan of their non-diabetic contemporaries, while that epigenome in diabetics in the nutritional environment of today is detrimental to lifespan.

## Background

Diabetes is a manifestation of the failure of the body to manage carbohydrate intake with aging and increased body weight. Over the past forty years there has been a progressive decrease in the defined upper limits of normal for fasting blood glucose categorizing more persons as diabetic, but even considering this changing diagnostic threshold there is a worldwide increase in the incidence and prevalence of diabetes mellitus, particularly type-2 diabetes [1-3]. Epigenetic mechanisms are likely involved, especially in the third world where obesity is a major, but not the only factor [4]. There are a wide variety of potential modulators of the human epigenome, which can be modified through methylation, X-inactivation, maternal imprinting, and microRNAs, as well as by environmental chemicals, toxins, caloric intake and dietary sugars [5-9]. In type-2 diabetes the early effects of maternal nutrition and exposure to carbohydrates may ‘set’ a course for adult disease [10-15]. Studies report that the nutrition of grandparents has been shown to affect future generations, particularly the male probands [8,16-19]. However, the most important modifying factors in type-2 diabetes in adults are excess caloric intake and decreased physical exercise. The Canadian Institutes of Health Research estimates that a North American child born in the year 2000 has one chance in three of being diabetic in his/her lifetime. The lifetime expenses associated with this diagnosis are considerable, and with a ‘baby boomer’ generation manifesting more diabetes as it ages, the societal cost for this metabolic disease in the United States will triple by the year 2034 [20,21].

Although type-2 diabetes is pathological in our current environment of surfeit, we hypothesize the disease is a manifestation of an ancient survival mechanism, for example, a manifestation of the ability of the human species to conservatively store and efficiently metabolize calories in times of hardship. In addition, we propose that those afflicted with type-2 diabetes are manifesting the epigenetic changes due to lifestyle (insufficient exercise, excess sugars), as well as by aging and genetic loading.

Our previous research has shown that changes in solar radiation, as seen in 11-year solar cycles, influence human lifespan [22]. The Sun varies in its radiation with approximately three years of more intense output (MAX) followed by approximately eight years of less radiation (MIN). We have shown in a study of death records of over 50 million persons in the USA that lifespan is decreased by 1.7 years on average in persons born and likely conceived at MAX of solar cycles [23]. We proposed that this finding may be due to the increased exposure of our genome to ultraviolet radiation (UVR), the most DNA-damaging wavelength reaching the Earth’s surface [24]. In our previously reported research we found that metabolic diseases, like diabetes, were suppressed during solar MAX relative to MIN; conversely, we found that major mental illness was more likely in those born during solar MAX [25,26]. In this paper we specifically investigate the modulation of lifespan of persons with type-2 diabetes with changes in the 11-year solar radiation cycle and seasonal variations in UVR.

## Results

**Figure 1:**
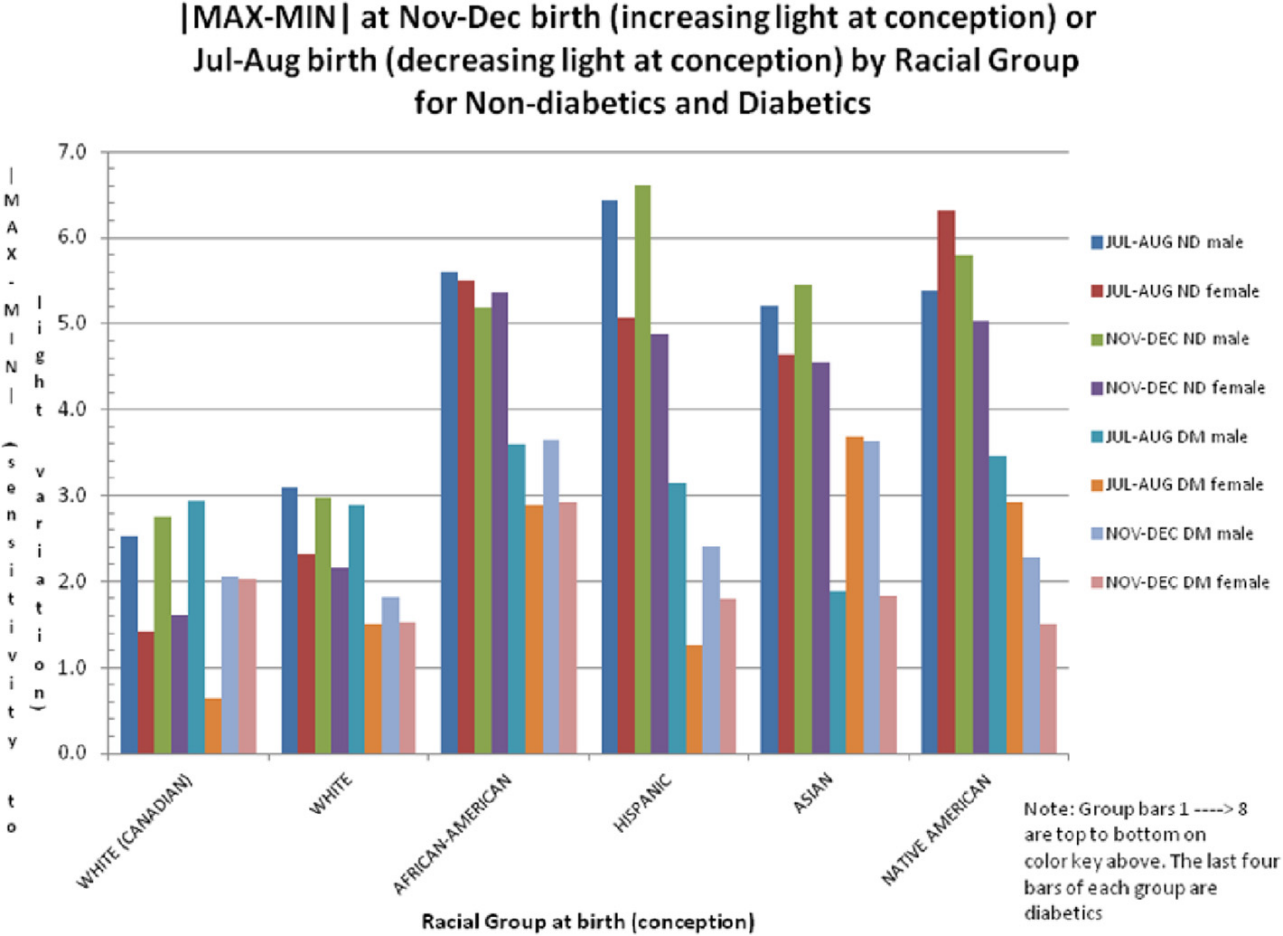
Examples of [max - min] for increasing and decreasing ultraviolet radiation (uvr) at conception by gender and by racial group for non-diabetics and diabetics.

**Figure 2:**
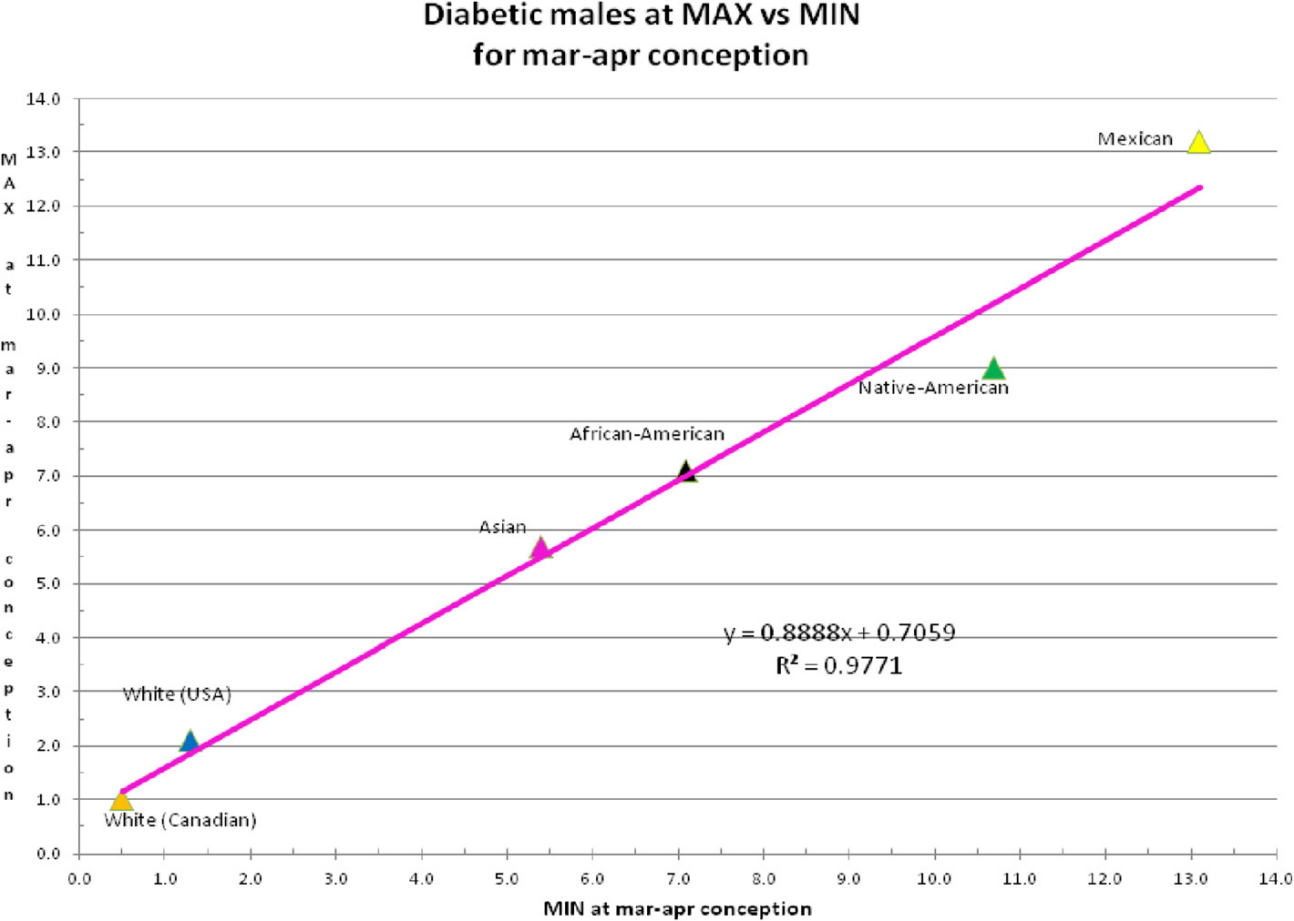
Example of male diabetic lifespan minus male non-diabetic lifespan at max versus min (in years) for various races in March and April conceptions.

**Figure 3:**
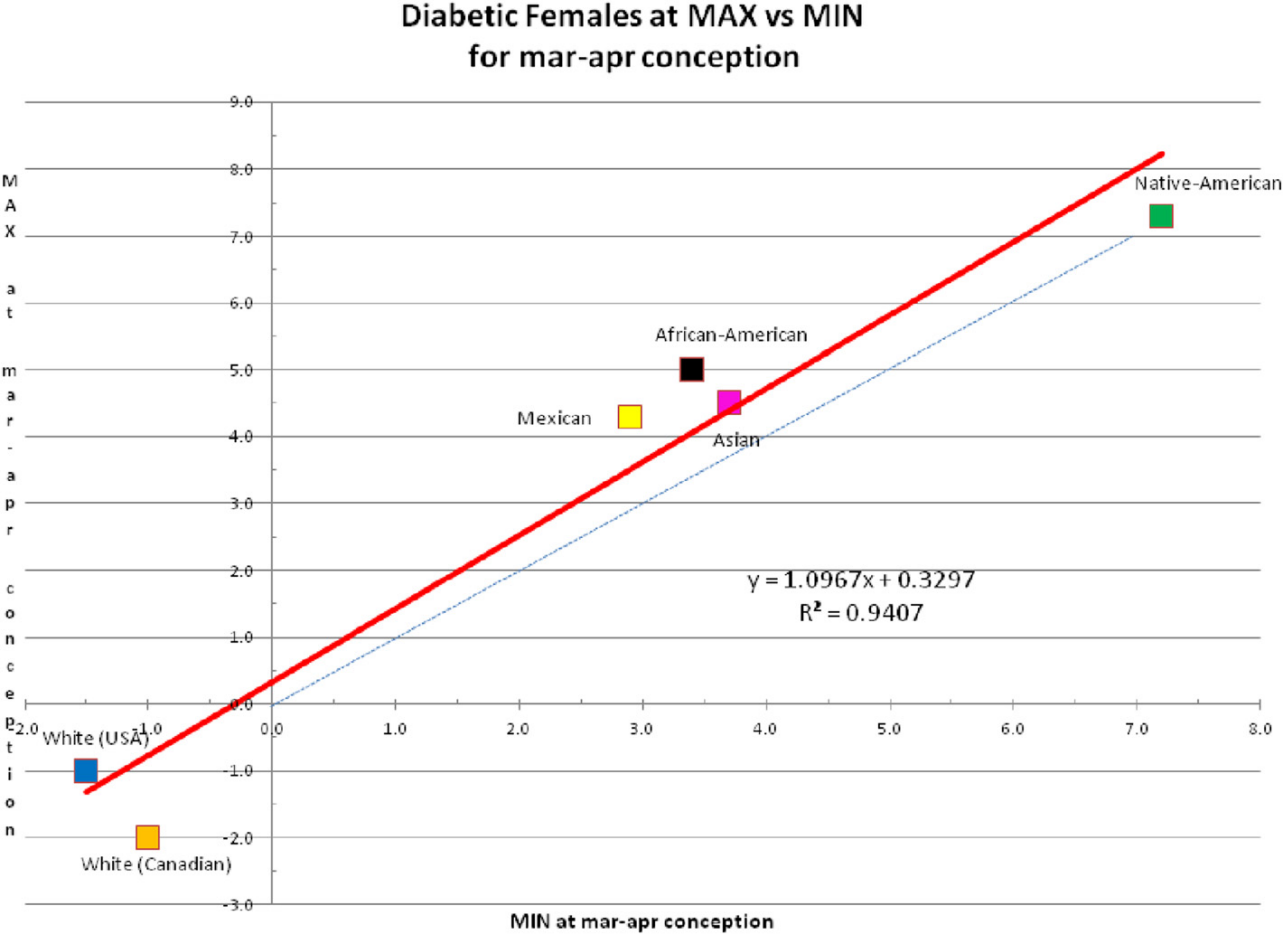
**Example of female diabetic lifespan minus female non-diabetic lifespan at max versus min (in years) for various races in March and April conceptions.** [See Appendix 2 in Additional file 1]. Dotted line is equipoise between the Y and X axis for reference.

We consider [MAX - MIN] for lifespan a metric for sensitivity (for example, responsiveness) to long-term changes in UVR. Figure 1 shows that the difference in lifespan between MAX and MIN is less in diabetes than in non-diabetics. In addition, ethnic minorities have a higher sensitivity, for example, a greater difference between MAX and MIN, than the white race. Also, females are generally less sensitive than males in their respective racial groups. Figure 2 (for males) and Figure 3 (for females) are examples from a single bimonthly period (March and April) plotting the difference between diabetic and non-diabetic lifespan at MAX versus at MIN for all racial groups. Note that the white and white Canadian group diabetic females in Figure 3 had a shorter lifespan than non-diabetics. This is similar to what we would see in today’s environment of surfeit in contrast to the ethnic minorities born largely before 1945.

As displayed at the bottom of Table 1 for MAX and Table 2 for MIN, the average years gained for males at MAX with increasing seasonal light at conception is greater than with decreasing seasonal light, and although there is more variation with females at MAX, there is little difference between the averages at increasing or decreasing light. At MIN however, males gain significantly more years above females when conceived in decreasing light.

**Table 1:** Male and female diabetics - regression equations and integral values at solar MAX

**Month of conception (All races)**	**Male diabetics regression EQ and (R**^**2**^**) at MAX**	**Integral of MAX years for MIN**	**Female diabetics regression EQ and (R**^**2**^**) at MAX**	**Integral of MAX years for MIN**	**Month of birth (All races)**
		**−5.0 to 10.0 (males)**		**−5.0 to 10.0 (females)**	
***Jan-Feb***	1.0663x + 0.0928 (0.9722)	*41.4*	1.1427x + 0.1749 (0.9590)	*45.5*	**Oct-Nov**
***Feb-Mar***	1.3704x + 0.5432 (0.9672)	*59.5*	1.3820x + 1.6371 (0.9902)	*76.4*	**Nov-Dec**
***Mar-Apr***	0.8888x + 0.7059 (0.9771)	*43.9*	1.0967x + 0.3297 (0.9407)	*46.1*	**Dec*-Jan***
***Apr-May***	0.9180x + 0.8914 (0.9964)	*47.8*	1.2347x - 0.0794 (0.9795)	*45.1*	***Jan-Feb***
***May-Jun***	0.9499x + 1.0587 (0.9846)	*51.5*	1.122x - 0.1454 (0.9414)	*39.9*	***Feb-Mar***
***Jun-*Jul**	1.067x - 0.3400 (0.9844)	*34.9*	1.0969x - 0.0983 (0.9213)	*39.7*	***Mar-Apr***
**Jul-Aug**	1.0918x - 0.8685 (0.9677)	*27.9*	0.6996x + 1.5517 (0.9797)	*49.5*	***Apr-May***
**Aug-Sep**	1.0383x - 0.2481 (0.9913)	*35.2*	1.0984x + 0.5254 (0.9656)	*49.1*	***May-Jun***
**Sep-Oct**	1.0019x + 0.1545 (0.9820)	*39.9*	1.0736x + 1.0948 (0.9689)	*56.7*	***Jun*-Jul**
**Oct-Nov**	1.2887x + 0.3252 (0.9787)	*53.2*	1.2419x + 1.6821 (0.9228)	*71.8*	**Jul-Aug**
**Nov-Dec**	0.9303x - 0.1257 (0.9508)	*33.0*	0.9222x + 0.0196 (0.9566)	*34.9*	**Aug-Sep**
**Dec-*Jan***	0.8933x + 0.2413 (0.9077)	*37.1*	0.8859x + 0.4906 (0.9375)	*40.6*	**Sep-Oct**
Average		*42.1*		*49.6*	
Sum		*505.3*		*595.3*	
SD		*9.4*		*12.8*	
**AVG (SD) years Increasing**		***46.5 (8.5)***		***48.7 (13.8)***	
**AVG (SD) years Decreasing**		***37.7 (8.6)***		***50.4 (12.9)***	

Note: Italics for months with increasing UVR.

**Table 2:** Male and female diabetics - regression equations and integral values at solar MIN

**Month of conception (All races)**	**Male diabetics regression EQ and (R**^**2**^**) at MIN**	**Integral of MIN years for MAX** **−5.0 to 10.0 (males)**	**Female diabetics regression EQ and (R**^**2**^**) at MIN**	**Integral of MIN years for MAX** **−5.0 to 10.0 (females)**	**Month of birth**
					**(All races)**
***Jan-Feb***	0.9117x + 0.0927 (0.9722)	*35.6*	0.8392x - 0.0337 (0.9590)	*31.0*	**Oct-Nov**
***Feb-Mar***	0.7058x - 0.2250 (0.9672)	*23.1*	0.7165x - 1.1636 (0.9902)	*9.4*	**Nov-Dec**
***Mar-Apr***	1.0993x - 0.6305 (0.9771)	*31.8*	0.8577x - 0.1375 (0.9407)	*30.1*	**Dec-*Jan***
***Apr-May***	1.0854x - 0.9453 (0.9964)	*26.5*	0.7933x + 0.1134 (0.9795)	*31.4*	***Jan-Feb***
***May-Jun***	1.0365x - 1.0068 (0.9846)	*23.8*	0.8391x + 0.2486 (0.9414)	*35.2*	***Feb-Mar***
***Jun*-Jul**	0.9225x + 0.4064 (0.9844)	*40.7*	0.8399x + 0.2315 (0.9213)	*35.0*	***Mar-Apr***
**Jul-Aug**	0.8864x + 0.9662 (0.9677)	*47.7*	0.9144x + 0.0114 (0.9655)	*34.5*	***Apr-May***
**Aug-Sep**	0.9547x + 0.2916 (0.9913)	*40.2*	0.8791x - 0.3822 (0.9656)	*27.2*	***May-Jun***
**Sep-Oct**	0.9801x - 0.0377 (0.9820)	*36.2*	0.9026x - 0.9296 (0.9689)	*19.9*	***Jun*-Jul**
**Oct-Nov**	0.7594x - 0.1394 (0.9787)	*26.4*	0.7431x - 1.1432 (0.9228)	*10.7*	**Jul-Aug**
**Nov-Dec**	1.022x + 0.4609 (0.9508)	*45.2*	1.0463x - 0.1893 (0.9749)	*36.4*	**Aug-Sep**
**Dec-*Jan***	1.0161x + 0.3772 (0.9077)	*43.8*	1.0582x - 0.3498 (0.9375)	*34.4*	**Sep-Oct**
Average		*35.1*		*27.9*	
Sum		*421*		*335*	
SD		*8.7*		*9.5*	
**AVG (SD) years Increasing**		***30.3 (7.0)***		***28.7 (9.7)***	
**AVG (SD) years Decreasing**		***39.9 (7.7)***		***27.2 (10.2)***	

Note: Italics for months with increasing UVR.

Data in Tables 1 and 2 for annual years of life gained are used in calculations resulting in Table 3, and subsequently, Table 4 (see Methods). For the convenience of the reader, month of birth (MOB) is easily converted to month of conception (MOC) by simply adding three months to the MOB; for example, a May birth has an August MOC.

**Table 3:** **Calculations using values from Tables**1**and**2


***For the MAX portion of solar cycles***	**Years**
Diabetic males at MAX in *increasing* UVR	241.8
Diabetic males at MAX in *decreasing* UVR	189.5
Diabetic females at MAX in *increasing* UVR	253.1
Diabetic females at MAX in *decreasing* UVR	262.8
***For the MIN portion of solar cycles***	**Years**
Diabetic males at MIN in *increasing* UVR	144.0
Diabetic males at MIN in *decreasing* UVR	195.0
Diabetic females at MIN in *increasing* UVR	137.0
Diabetic females at MIN in *decreasing* UVR	128.5

**Table 4:** Calculations using values from Table 3


**For*decreasing*UVR at conception**	Ratio years gained at MAX by male diabetics/female diabetics
189.5/ 262.8 =	0.72
	Ratio years gained at MIN by male diabetics/female diabetics
195.0/ 128.5 =	1.52
**For*increasing*UVR at conception**	Ratio years gained at MAX by male diabetics/female diabetics
241.8/ 253.1 =	0.96
	Ratio years gained at MIN by male diabetics/female diabetics
144.0/ 137.0 =	1.05

**Figure 4:**
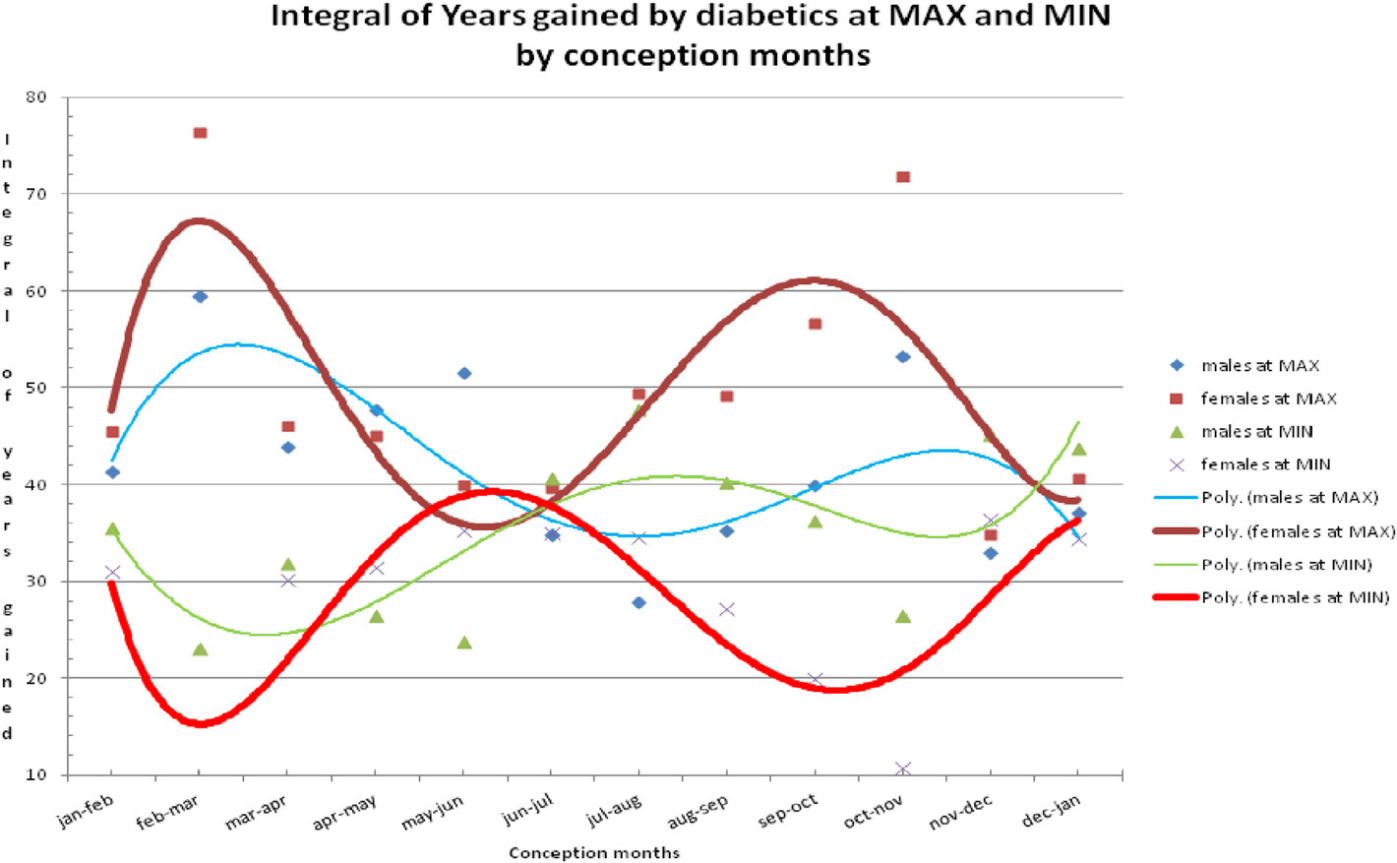
**Trend lines of integral years gained by gender at max and min by months of conception for diabetics who were born before year 1945.** poly, 3rd degree polynomial trend lines of data from Tables 1 and 2 for male and female diabetics.

Figure 4 displays the monthly data from Tables 1 and 2 showing that most of the variation in lifespan occurs at the equinoxes when UVR varies the most rapidly. Note in Figure 4 that males have a bilobed pattern for MAX and MIN at the fall equinox while females have a single lobe. Both males and females have a single lobe at the spring equinox. The reason for these differences is not clear. The least variation in lifespan occurs at the solstices in both genders. We found that the weighted (72% MIN and 28% MAX) average lifespan years gained by male diabetics over male non-diabetics was 6.1 years [see Appendix 2 in Additional file 1], whereas that difference in females was only 2.3 years. Therefore, diabetic males gained (6.1 - 2.3 =) 3.8 years over diabetic females on average.

The complexity of this study is related to several overlapping variables that include:

1) UVR intensity and variation→ where variation is likely more important than intensity in activating diabetes.

2) Seasons and solar cycles→ where seasons appear more important than solar cycle variation in affecting diabetes, but appear to be independent of vitamin D (see Discussion).

3) Being conceived and likely born *before* 1945 or *after* 1945→ nutritional environments change from poorer nutrition to caloric excess between the two periods.

4) Diabetics and non-diabetics→ in times of hardship persons who live longer are more likely to express diabetes (from aging itself); in times of plenty diabetics live shorter lives.

5) Whites and ethnic minorities→ the more melanin, the more sensitive to changes in solar cycle UVR; for example, long-term changes are more important than short-term (seasonal) changes (see Figure 1).

6) Males and females→ males are more sensitive to variation in UVR; women are more canalized with regard to caloric retention and are less sensitive.

Table 5 concisely summarizes these relationships.

**Table 5:** Summary of findings related to ultraviolet radiation (uvr) variations

**Lower sensitivity (smaller MAX - MIN)**	**Higher sensitivity (larger MAX - MIN)**
Diabetics	Non-diabetics
More genetic	More epigenetic
White race	Ethnic minority races
Ethnic minority diabetics	Ethnic minority non-diabetics
Female ethnic minority diabetics	Male ethnic minority diabetics
Diabetic females less affected by seasonal changes in UVR, slightly benefit at MAX	Diabetic males benefit by decreasing seasonal UVR at conception, and at MIN

## Discussion

This paper presents evidence that UVR, which varies in intensity with solar cycles and seasons, modulates the lifespan of type-2 diabetics born before 1945. Using the death records of approximately 829,000 diabetics born before 1945, we found that ethnic minority diabetics lived significantly longer than their non-diabetic minority contemporaries. In contrast, in the last decades of the 20th century Native Americans with a high prevalence of obesity and diabetes have a shorter lifespan [27]. However, the white diabetic females born prior to 1945 revealed what we would expect today, for example, shorter lives most probably due to vascular complications. We suspect that the difference in lifespan between whites and ethnic minorities was due to poorer nutrition and hard physical work in the latter groups; indeed, most of human history was a continuous struggle to obtain food [28]. Many ethnic minorities had fled from famine in Europe and Asia and took laborious jobs in the United States, but despite the USA being ‘a land of plenty’, nutrition was a significant problem manifested by pellagra (niacin deficiency) at least through the 1940s [29].

Those with epigenetic alterations to increase metabolic efficiency and caloric storage would have a survival advantage in an adverse environment. While the hunter-gatherers of pre-historical humans generally had adequate nutrition, throughout human history there have been periodic famines in all continents with, in some cases, the loss of millions of lives [30-33]. In our study ethnic minorities, had a modest caloric intake readily metabolized by hard physical labor [34,35]. As discussed by Lane, people for the last 6,000 years have struggled to get something to eat, and even in modern times many in the world go without adequate nutrition [28]. Obesity was subsequently less prevalent than in the post 1950 to the present era.

There is increasing evidence that early life events have effects that modulate the adult expression of disease. We hypothesize that rapidly changing UVR is most influential at conception/early gestation, particularly at the equinoxes, and alters epigenetic expression in diabetes. Rapidly decreasing UVR at conception, for example, at the autumnal equinox, was disadvantageous to the white race (and those in other races of higher social status) possibly because of good nutrition and less physically demanding jobs where activation of ‘frugal’ epigenes only predisposes to obesity [36]. Women in general are at a particular disadvantage probably due to a canalized tendency to retain weight for pregnancy and due to the effect of estrogens; however, rapidly increasing UVR at conception, for example, at or around the vernal equinox, may be beneficial to women due to the suppression of a calorie-conservative epigenome and may also be useful to men who are genetically predisposed to become overweight or who are underactive. A Ukrainian study has recently showed that type 2 diabetes is more common in those born in April (for example, conception in July with decreasing UVR), and is less common in those born in November and December (for example, conception in February and March with increasing UVR) [37].

The above observations suggest that increased skin melanin is associated with an *increased* sensitivity to ambient UVR as seen in Figure 1. One might have expected the reverse. However, melanin is an adaptation to increased UVR intensity, and more sensitivity to any variation in UVR may be necessary and encoded in the human genome over millennia [24].

One can speculate about how UVR affects an embryo *in utero* without direct exposure to UVR. A comprehensive review of how the skin senses the environment was recently published [38]. With the skin sharing an ectodermal origin with the central nervous system, it is not surprising to find shared neuroregulatory compounds in both tissues, including serotonergic, melatoninergic, catacholinergic, opioidergic, and the hormone vitamin D, among several others. Circulating inflammatory cytokines, chemokines, photo-oxidation products, and nitric oxide may also play a role [39-41].

There are recent reports that hypovitaminosis D is associated with a higher incidence of diabetes [42-44]. This is especially true for those with more skin pigmentation [45]. Those persons working in agrarian jobs would spend much more time outdoors, and by increasing their vitamin D level, could mitigate or delay the expression of diabetes. The literature supports adequate vitamin D increasing insulin sensitivity [46]. This may be an additional reason for the greater lifespan of those with diabetes around the turn of the last century as well as greater physical activity and less obesity. Vitamin D is important in modifying a human epigenome involved with many adult disorders [47]. However, as Figure 4 shows, in this study of our cohort of diabetics there is no difference between the summer and winter solstice in added life years and the major variation in lifespan occurs at the equinoxes. Any effect of vitamin D on lifespan would therefore extend beyond the annual seasonal cycle. Variations in food supply occurring cyclically over years, would give a survival advantage to those individuals with an increased ability to store calories and vitamin D in adipose tissue.

The heterogeneity of our sample results in some persons that benefit, and some who are harmed, by activating or suppressing the metabolically sensitive epigenome, as is evident in Figure 4. Table 5 summarizes observations from Tables 1 and 2.

We are aware that type-2 diabetes is a heterogeneous disease affecting many organ systems and contributing to death in a various ways. However, we wanted to study specifically how UVR affects lifespan, not any other clinical aspects of this disease. Separating the effects of long-term solar-cycle radiation from that of seasonal (annual) radiation was challenging. We also probably did not detect more than one-third of the diabetics in our database, but for those who were captured, diabetes must have played a significant role to be entered on a death certificate, and therefore, these cases were likely to be the most severe.

We do not have comparable death data of persons who were born and died post-1945, and another half-century will pass before that data are available. However, it is already clear that there is an epidemic of type-2 diabetes worldwide. Women are particularly adversely affected in our current environment [48,49]. As we have shown, diabetic white women were losing lifespan even before 1945. Some of these findings are obviously due to a rising standard-of-living in highly developed countries, for example, better food supplies and an increased use of mechanical devices in lieu of manual labor.

Reports suggest that white race northern Europeans may have developed a partial resistance to diabetes over the centuries, becoming adapted to a lactose-rich diet through farming/animal husbandry, and to rapidly changing light at higher latitude through natural selection [50,51].

Migration is probably another important factor in the increasing incidence of type-2 diabetes. People who are indigenous to a high-intensity, low-variation photonic environment, like those born near or on the Equator, who then migrate to higher latitude, may be more susceptible to the trigger of decreasing UVR at conception (especially at the autumnal equinox), and along with a nutrient-rich lifestyle, more readily acquire type-2 diabetes. This may explain, in part, the increased prevalence of type-2 diabetes in immigrants to northern Europe and to North America [52]. Once adjusted for ethnicity and socio-demographic variables, migration alone may not be crucial to health outcomes [53]. This opens the possibility that other factors, yet to be fully studied, like solar cycle variations of UVR and geomagnetic forces, modulate our epigenome [54].

One way of prospectively confirming the hypothesis in this paper would be to use ‘knockout’ mice predisposed to diabetes and see if increasing or decreasing UVR exposure in the early days of gestation (for mice, possibly as little as a 2-day exposure out of a 19-day gestation) to see if the expression of diabetes occurs earlier or later in the animal’s lifetime. Using UVR to treat humans with a genetic predisposition to diabetes must await the results of prospective animal studies along with an increased knowledge of our epigenome [55].

## Conclusions

The significant findings of this research are summarized as follows:

•The effects of UVR modulate human lifespan in diabetics; for example, both solar and seasonal cycles affect the human epigenome. We believe that UVR has a more profound effect at conception/ early gestation, but may still affect adults, albeit more weakly as in seasonal affective disorder when an increased appetite and weight gain can be a problem.

•The difference between lifespan at MAX and MIN serves as a surrogate for sensitivity (or responsiveness) to long-term variation in UVR. The differences in lifespan between months would analogously apply for short-term variation.

•Ethnic minority diabetics, who were born before the year 1945, lived slightly longer than non-diabetics of the same period. Our data analysis supports the hypothesis that epigenetic effects that conserve energy benefit survival in persons living with marginal nutrition and exposed to hard physical labor.

•Diabetic women have canalized metabolic conservation more effectively than men, primarily for fertility/pregnancy, and are less affected by UVR than men, but consequently are more susceptible to weight gain in times of surfeit.

•The hypothesis that we might modulate the phenotypic expression of type-2 diabetes by using UVR; for example, optogenetics, is eminently testable.

•Other forces associated with our variable star, like geomagnetic fields, may also be playing a role in modulating our epigenome and are areas for future research.

## Methods

### Data

Data were obtained from the National Center for Health Statistics (NCHS) for deaths in all 50 states and the District of Columbia from 1979 to 2005 - a total of 58,733,243 deaths. The data were de-identified to preserve confidentiality with only month and year of birth obtained. Data used in this study included sex, state of birth, date of birth (month and year), date of death (month and year), and race. Since data on Hispanic origin was not recorded from 1979 to 1988, race categories used for this study included white, African-American, Native-American/Alaska Native, Asian/Pacific Islander races. White Canadians in the study were born in Canada but lived and died in the United States. From 1989 to 2005 we had access to a Mexican (Hispanic) cohort. These were persons born largely in Mexico but lived and died in the United States. For this study the white race (N = 50,778,214) was the largest group. The sample contained 51% males and 49% females. Approximately 90% of the persons in the database were born in or before the year 1945 and would have died between 1979 and 2005. The average age at death was 71. We did not age match controls, but given the sample size, and by randomization, we derived a reasonable control set. The persons in our database lived in challenging times, which included a Great Depression occurring between two World Wars in an agrarian/ industrial economy.

### Solar data

The Sun is a variable star which varies its electromagnetic and plasma energy output in an approximate 11-year cycle (varying 9 to 14 years). A measure of this energy output is the sunspot number. Sunspots are magnetic storms on the Sun’s surface that indicate increased fusion activity and their number correlates positively with the intensity of solar radiation including ultraviolet wavelengths. UVR is an important source of Earth’s energy and despite that only 1 percent of the sun’s energy is emitted at UVR wavelengths between 200 and 300 nm, UVR accounts for nearly 20 percent of the variation in total irradiance. A recent study revealed that a 5% variation in ground-level UVR (though not related to solar cycles) produces a 24% variation in effects on DNA [56-59]. The average annual number of sunspots was collected from the NOAA (National Oceanic and Atmospheric Administration) Web site and the three MAX years (approximately 28%) of each of the past twelve cycles was obtained to be compared with the remaining MIN years (approximately 72%) [60]. The average annual sunspot number for the past 250 years was found to be 49; for the past 60 years the average is 107.5; for the most powerful cycles (sunspots >135), the average is 154, about three times the 250-year average. The time period of our study comprised 13 solar cycles [25].

### Statistical methodology

Birth year data were grouped by solar MAX or MIN and were defined as follows: the year before and the year after the peak years were defined as the Maximum Solar Period (MAX); the years before and after each three-year MAX cycle were grouped as Minimum Solar Period (MIN). We selected diabetics using ICD-9 codes recorded as cause of death. Total diabetics plus non-diabetics equaled approximately 54 million, of which 28% were in the MAX group, which equaled approximately 15 million cases Diabetics accounted for 1.5% of the total all-racial set, and those born in a MAX period accounted for 226,442 diabetics. These were matched with an equal number of randomly selected non-diabetics for further analysis, serving as control. Table 6 is an example of the white race from Additional file 2: Appendix 1 (Table S1 -S6) and gives lifespan statistics for diabetics and non-diabetics by bimonthly groups. Table 7 lists the statistics of each racial group. Table 8 lists the total of diabetics and non-diabetics by racial group.

**Table 6:** **Displays an example from Additional file****2****: Appendix 1 for two bimonthly periods for increasing UVR at conception and for decreasing UVR at conception for the white race (a similar analysis was done for all 12 bimonthly sets for each racial group**

**White**	**Average MAX-MIN**	**Average MAX-MIN**
	**July-August Births (years)**	**November-December Births (years)**
*All* male	**−3.167**	**−3.141**
(White)	avg^a^ age MAX:	avg age MAX:
Non-diabetic	67.4	67.8
	avg age at MIN:	avg age at MIN:
	70.6	71.0
	n max = 815,000	n max = 756,000
	n min = 795,000	n min = 726,000
	standard error = 0.03	standard error = 0.03
	*p* <0.0001	*p* <0.0001
*All* female	**−2.479**	**−2.360**
Non-diabetic	avg age MAX:	avg age MAX:
	74.7	75.2
	avg age at MIN:	avg age at MIN:
	77.2	77.5
	n max = 759,000	n max = 708,000
	n min = 796,000	n min = 730,000
	standard error = 0.03	standard error = 0.03
	*p* <0.0001	*p* <0.0001
*Diabetic* male	**−2.610**	**−2.057**
	avg age MAX:	avg age MAX:
	68.7	69.6
	avg age at MIN:	avg age at MIN:
	71.3	71.6
	n max = 9,756	n max = 9,117
	n min = 9,783	n min = 9,150
	standard error = 0.19	standard error = 0.20
	*p* <0.0001	*p* <0.0001
*Diabetic* female	**−2.074**	**−1.789**
	avg age MAX:	avg age MAX:
	73.6	74.0
	avg age at MIN:	avg age at MIN:
	75.4	75.8
	n max = 11,493	n max = 11,053
	n min = 12,391	n min = 11,936
	standard error = 0.16	standard error = 0.17
	*p* <0.0001	*p* <0.0001
Equivalent months of conception	OCT-NOV (decreasing UVR)	FEB-MAR (increasing UVR)

^a^avg, average; abbr, abbreviation defined

**Table 7:** Sample total by race

**Race**	**Count**	**Percent**	**Mean age**	**SD**	**Median age**
White	45,714,048	84.1	71.9	18.6	76
White (Canadian)	450,760	0.8	78.5	13.4	81
African-American	6,747,125	12.4	62.1	23.1	67
Asian	672,286	1.2	66.2	22.3	72
Native American	249,169	0.5	57.3	24.5	62
Hispanic (Mexican)	532,151	1.0	63.4	23.3	69
**Totals**	**53,914,779**	**100**	**70.5**		**75**

**Table 8:** Total diabetics and non-diabetics by racial group

**Race**	**Count of diabetics**	**Count of non-diabetics**
White	646,808	45,070,000
African-American	146,598	6,600,290
Native American	6,951	242,208
Asian	9,903	662,383
White Canadian	5,766	444,993
Mexican	12,705	519,430
**Totals**	**828,731**	**53,539,304**^**a**^

^a^The totals in Tables 7 and 8 do not agree by 0.84% due to the fact that in some cases diagnosis was not recorded.

In this paper we report both birth and conception months and we consider the month of conception to be 10 months (approximately 39 weeks) earlier than birth month. We frequently refer to conception months as we believe that the conceptus is more sensitive to the effects of environmental UVR than at the time of birth.

### Data analysis

We subtracted the age-at-death of non-diabetics from the age-at-death of diabetics for all races for months of conception (10 months earlier than months of birth), and for both genders for MAX and MIN of solar cycles. These data, derived from Additional file 2: Appendix 1 (which only shows a single bimonthly sample out of a full year of data), are displayed in Additional file 1: Appendix 2 [see Table S1-S6]. The data from Additional file 1: Appendix 2 were then plotted for each set of months (bimonthly to increase N), MAX on the Y-axis, MIN on the X-axis (see Figures 2 and 3). Regression equations were calculated by Excel and were integrated with *Mathematica* version 7.0 from −5.0 to 10.0 years (a reasonable range seen in the regressions) to calculate the overall number of years of life gained (or lost) by diabetics over non-diabetics. We performed this procedure for both MAX and MIN (by reversing the axes) and the results of lifespan years gained or lost are displayed in Tables 1 and 2 in the 3rd and 5th columns. The high R^2^ (all 24 equations, 12 for MAX and 12 for MIN, was >0.90) suggests a strong relationship between radiant energy at MAX and MIN and the lifespan difference between diabetics and non-diabetics. The integrals of the linear regression lines from Tables 1 and 2 were plotted on the Y-axis with months of conception on the X-axis, for MAX and MIN and for males and females as shown in Figure 4.

When months of the year with increasing light are compared with those months with decreasing at both MAX and MIN, we must take into account that the solstices occur about 10 days (approximately 16% of a two-month period) before the end of the month. Further calculations were performed using the data from Tables 1 and 2, an example of which follows for the MAX portion of solar cycles:

Diabetic Males at MAX in increasing UVR: (0.16) (Dec-Jan) + (Jan-Feb) + (Feb-Mar) + (Mar-Apr) + (Apr-May) + (0.84) (May-Jun) = (0.16) (37.1) + (41.4) + 59.5 + 43.9 + 47.8 + (0.84) (51.5) = 241.8 years

The Table 3 summarizes calculations of integral years gained by diabetics. Table 4 shows the calculations using the data in Table 3.

Since MIN comprises approximately 72% of a solar cycle and MAX approximately 28%, then a weighted average using values from Table 4:

Male /Female diabetic ratio = (0.72) (1.52) + (0.28) (0.72) = 1.09 + 0.20 = **1.30** for *decreasing* UVR

Similarly,

Male/ Female diabetic ratio = (0.72) (1.05) + (0.28) (0.96) = 0.76 + 0.27 = **1.02** for *increasing* UVR

Therefore, the male/female ratio of years gained by diabetics in *decreasing* UVR/ *increasing* UVR = 1.30/ 1.02 = **1.27**

Note that the above ratios indicate *relative* effects and do not translate directly to differences in lifespan as measured in years.

### Ethics statement

As the data obtained were de-identified, and as there were no direct therapeutic interventions of persons, we declare no ethical conflicts in our study.

### Study strengths

The large death records database from the entire United States gives high statistical power. We did not have to use life expectancy tables as we had the actual time of birth and death of each case.

### Study limitations

The use of death certificates by the database for diagnosis is a limitation in this study. However, as stated previously, those listed as diabetic were the most seriously afflicted so the diagnosis would probably not be in doubt. The small number of type-1 diabetics (usually approximately 5 to 10%) mixed in the sample would not significantly alter our conclusions.

### How this study contributes to new knowledge

We believe this study supports the effect of UVR in modulating the human epigenome in type-2 diabetes.

## Abbreviations

MOB: month of birth; MOC: month of conception; MAX: peak of solar cycle; MIN: trough of solar cycle; UVR: ultraviolet radiation.

## Competing interests

The authors declare that they have no competing interests.

## Authors’ contributions

The authors contributed equally to this work. GED wrote the manuscript, WEL analyzed the data and performed statistical work. All authors read and approved the final manuscript.

## Supplementary Material

Additional file 1: Appendix 2 **(A)**: RESULTS for MALES of Step 1 in Sequence of analysis [diabetic minus non-diabetic lifespan]. MOC = month of conception; MOB = month of birth. **(B)**: RESULTS for FEMALES of Step 1 in Sequence of analysis [diabetic minus non-diabetic lifespan]. MOC = month of conception; MOB = month of birth. Click here for file

Additional file 2: Appendix 1. Table S1 [MAX – MIN] for White Non-diabetics & Diabetics by Increasing or Decreasing light at Birth (or Conception). **Table S2**: [MAX – MIN] for Asian Non-diabetics & Diabetics by Increasing or Decreasing light at Birth (or Conception). **Table S3**: [MAX – MIN] for White (Canadian) Non-diabetics & Diabetics by Increasing or Decreasing light at Birth (or Conception). **Table S4**: [MAX – MIN] for Hispanic Non-diabetics & Diabetics by Increasing or Decreasing light at Birth (or Conception). **Table S5**: [MAX – MIN] for African-American Non-diabetics & Diabetics by Increasing or Decreasing light at Birth (or Conception). **Table S6**: [MAX – MIN] for Native American Non-diabetics & Diabetics by Increasing or Decreasing light at Birth (or Conception). Click here for file
