# Supplementary material for: Variation in ultraviolet radiation and diabetes: evidence of an epigenetic effect that modulates diabetics’ lifespan
Source: Clin Epigenetics. 2013 Apr 2;5(1):5. doi: 10.1186/1868-7083-5-5 (PMC3639074; doi:10.1186/1868-7083-5-5)
Supplement: Additional file 1: Appendix 2 — (A): RESULTS for MALES of Step 1 in Sequence of analysis [diabetic minus non-diabetic lifespan]. MOC = month of conception; MOB = month of birth. (B): RESULTS for FEMALES of Step 1 in Sequence of analysis [diabetic minus non-diabetic lifespan]. MOC = month of conception; MOB = month of birth. [file 1868-7083-5-5-S1.pdf]

# APPENDIX 2 (A): RESULTS for MALES of Step 1 in Sequence of analysis [diabetic minus non-diabetic lifespan]

## MOC = month of conception; MOB = month of birth

| | | | MALES ONLY | | | MAX DATA | | | | | | | | *race stats* | |
|---|---|---|---|---|---|---|---|---|---|---|---|---|---|---|---|
| **MOC** | **dec-jan** | **jan-feb** | **feb-mar** | **mar-apr** | **apr-may** | **may-jun** | **jun-jul** | **jul-aug** | **aug-sep** | **sep-oct** | **oct-nov** | **nov-dec** | **mean** | **median** | **sd** |
| **RACE** | | | | | | | | | | | | | | | |
| *White* | 1.5 | 1.8 | 1.8 | 2.1 | 2.0 | 1.9 | 1.3 | 1.5 | 1.6 | 1.6 | 1.3 | 1.7 | **1.7** | 1.7 | 0.3 |
| *White (Canadian)* | -0.9 | -0.6 | 0.9 | 1.0 | 1.2 | 1.2 | -1.1 | -1.1 | -0.2 | -0.3 | -0.6 | -1.5 | ***-0.2*** | *-0.5* | *1.0* |
| *Black* | 8.0 | 8.3 | 7.5 | 7.1 | 7.1 | 6.4 | 6.6 | 6.9 | 6.8 | 1.6 | 7.9 | 7.5 | ***6.8*** | *7.1* | *1.7* |
| *Asian* | 5.9 | 7.4 | 6.8 | 7.6 | 6.0 | 7.2 | 5.9 | 4.7 | 6.2 | 7.6 | 8.4 | 6.1 | ***6.7*** | *6.5* | *1.0* |
| *Mexican* | 14.4 | 13.0 | 13.9 | 13.2 | 12.9 | 12.4 | 12.6 | 13.4 | 13.8 | 13.3 | 13.9 | 13.8 | ***13.4*** | *13.4* | *0.6* |
| *Native American* | 8.7 | 11.4 | 12.1 | 9.0 | 10.1 | 10.7 | 10.6 | 9.3 | 9.6 | 9.5 | 10.1 | 9.4 | ***10.0*** | *9.9* | *1.0* |
| **mean** | ***6.3*** | ***6.9*** | ***7.2*** | ***6.7*** | ***6.6*** | ***6.6*** | ***6.0*** | ***5.8*** | ***6.3*** | ***5.6*** | ***6.8*** | ***6.2*** | 6.4 | | |
| **median** | *7.0* | *7.9* | *7.2* | *7.4* | *6.6* | *6.8* | *6.3* | *5.8* | *6.5* | *4.6* | *8.2* | *6.8* | | 6.8 | |
| **sd** | *5.5* | *5.3* | *5.3* | *4.5* | *4.5* | *4.5* | *5.2* | *5.3* | *5.1* | *5.4* | *5.5* | *5.5* | | | 0.5 |
| ***month stats*** | | | | | | | | | | | | | | | |
| **MOB** | sep-oct | oct-nov | nov-dec | dec-jan | jan-feb | feb-mar | mar-apr | apr-may | may-jun | jun-jul | jul-aug | aug-sep | | | |

| | | | MALES ONLY | | | MIN DATA | | | | | | | | *race stats* | |
|---|---|---|---|---|---|---|---|---|---|---|---|---|---|---|---|
| **MOC** | **dec-jan** | **jan-feb** | **feb-mar** | **mar-apr** | **apr-may** | **may-jun** | **jun-jul** | **jul-aug** | **aug-sep** | **sep-oct** | **oct-nov** | **nov-dec** | **mean** | **median** | **sd** |
| **RACE** | | | | | | | | | | | | | | | |
| *White* | 1.3 | 1.8 | 0.6 | 1.3 | 1.4 | 1.4 | 1.2 | 1.4 | 1.4 | 1.2 | 0.7 | 1.5 | **1.3** | 1.4 | 0.3 |
| *White (Canadian)* | -0.4 | 0.2 | 0.1 | 0.5 | 0.5 | 0.0 | -0.2 | 0.5 | 0.3 | 0.4 | -0.2 | -0.4 | ***0.1*** | *0.2* | *0.3* |
| *Black* | 6.8 | 6.8 | 6.1 | 7.1 | 6.5 | 5.9 | 5.8 | 6.0 | 6.4 | 1.2 | 5.9 | 6.8 | ***5.9*** | *6.3* | *1.6* |
| *Asian* | 6.1 | 5.7 | 4.9 | 6.6 | 5.1 | 6.0 | 6.4 | 6.3 | 6.4 | 6.6 | 5.1 | 6.3 | ***6.0*** | *6.2* | *0.6* |
| *Mexican* | 14.2 | 12.8 | 8.7 | 13.1 | 13.3 | 12.6 | 12.7 | 13.2 | 13.1 | 13.4 | 10.7 | 14.1 | ***12.7*** | *13.1* | *1.5* |
| *Native American* | 12.6 | 10.9 | 8.6 | 10.7 | 10.1 | 9.3 | 9.6 | 9.1 | 10.2 | 9.9 | 8.1 | 14.1 | ***10.3*** | *10.0* | *1.7* |
| **mean** | ***6.8*** | ***6.4*** | ***4.8*** | ***6.6*** | ***6.2*** | ***5.9*** | ***5.9*** | ***6.1*** | ***6.3*** | ***5.5*** | ***5.1*** | ***7.1*** | 6.0 | | |
| **median** | *6.5* | *6.3* | *6.9* | *6.9* | *5.8* | *6.0* | *6.1* | *6.2* | *6.4* | *3.9* | *5.5* | *6.6* | | 6.2 | |
| **sd** | *5.8* | *4.9* | *3.8* | *5.0* | *4.9* | *4.7* | *4.9* | *4.8* | *4.9* | *5.4* | *4.2* | *6.1* | | | 0.6 |
| ***month stats*** | | | | | | | | | | | | | | | |

## APPENDIX 2 (B): RESULTS for FEMALES of Step 1 in Sequence of analysis [diabetic minus non-diabetic lifespan]

## MOC = month of conception; MOB = month of birth

| | | | FEMALES ONLY | | | MAX DATA | | | | | | | | race stats | |
|---|---|---|---|---|---|---|---|---|---|---|---|---|---|---|---|
| MOC | dec-jan | jan-feb | feb-mar | mar-apr | apr-may | may-jun | jun-jul | jul-aug | aug-sep | sep-oct | oct-nov | nov-dec | mean | median | sd |
| RACE | | | | | | | | | | | | | | | |
| *White* | -1.0 | -1.0 | -1.2 | -1.0 | -1.3 | -1.3 | -1.2 | -1.3 | -1.2 | -1.2 | -1.1 | -1.1 | ***-1.2*** | *-1.2* | *0.1* |
| *White (Canadian)* | -0.9 | -1.3 | -5.4 | -2.0 | -2.1 | -2.5 | -2.7 | -2.8 | -2.5 | -2.1 | -2.2 | -2.2 | ***-2.4*** | *-2.2* | *1.1* |
| *Black* | 4.9 | 4.9 | 5.2 | 5.0 | 4.2 | 4.0 | 4.2 | 4.2 | 4.7 | 5.0 | 5.6 | 5.0 | ***4.7*** | *4.9* | *0.5* |
| *Asian* | 3.5 | 4.7 | 5.6 | 4.5 | 4.7 | 5.5 | 5.2 | 5.3 | 4.8 | 3.9 | 4.5 | 3.8 | ***4.7*** | *4.7* | *0.7* |
| *Mexican* | 2.9 | 2.8 | 4.3 | 4.3 | 3.0 | 1.2 | 1.0 | 3.4 | 4.2 | 4.2 | 4.6 | 2.4 | ***3.2*** | *3.2* | *1.2* |
| *Native American* | 7.9 | 9.8 | 9.2 | 7.3 | 9.3 | 6.8 | 5.4 | 5.5 | 8.4 | 8.9 | 9.0 | 7.6 | ***7.9*** | *8.2* | *1.5* |
| mean | ***2.9*** | ***3.3*** | ***3.0*** | ***3.0*** | ***3.0*** | ***2.3*** | ***2.0*** | ***2.4*** | ***3.1*** | ***3.1*** | ***3.4*** | ***2.6*** | 2.8 | | |
| median | *3.2* | *3.8* | *4.8* | *4.4* | *3.6* | *2.6* | *2.6* | *3.8* | *4.5* | *4.1* | *4.6* | *3.1* | | 4.0 | |
| sd | *3.4* | *4.2* | *5.3* | *3.7* | *4.2* | *3.8* | *3.5* | *3.5* | *4.1* | *4.1* | *4.3* | *3.7* | | | 0.5 |
| *month stats* | | | | | | | | | | | | | | | |
| MOB | sep-oct | oct-nov | nov-dec | dec-jan | jan-feb | feb-mar | mar-apr | apr-may | may-jun | jun-jul | jul-aug | aug-sep | | | |

| | | | FEMALES ONLY | | | MIN DATA | | | | | | | | race stats | |
|---|---|---|---|---|---|---|---|---|---|---|---|---|---|---|---|
| MOC | dec-jan | jan-feb | feb-mar | mar-apr | apr-may | may-jun | jun-jul | jul-aug | aug-sep | sep-oct | oct-nov | nov-dec | mean | median | sd |
| RACE | | | | | | | | | | | | | | | |
| *White* | -1.4 | -1.3 | -1.7 | -1.5 | -1.5 | -1.5 | -1.7 | -1.6 | -1.5 | -1.7 | -1.8 | -1.5 | ***-1.6*** | *-1.5* | *0.1* |
| *White (Canadian)* | -1.9 | -1.7 | -5.1 | -1.0 | -1.5 | -1.9 | -1.7 | -2.0 | -2.5 | -2.6 | -2.9 | -2.5 | ***-2.3*** | *-2.0* | *1.0* |
| *Black* | 4.2 | 4.2 | 2.8 | 3.4 | 3.4 | 3.0 | 2.8 | 3.1 | 3.3 | 4.1 | 3.0 | 4.1 | ***3.5*** | *3.4* | *0.6* |
| *Asian* | 5.2 | 4.6 | 2.9 | 3.7 | 4.0 | 4.5 | 4.2 | 5.1 | 5.1 | 1.4 | 3.6 | 4.8 | ***4.1*** | *4.4* | *1.1* |
| *Mexican* | 2.5 | 3.3 | 1.2 | 2.9 | 3.3 | 2.7 | 2.0 | 2.7 | 2.6 | 2.5 | 0.8 | 2.2 | ***2.4*** | *2.6* | *0.8* |
| *Native American* | 7.6 | 7.4 | 5.6 | 7.2 | 7.1 | 6.2 | 5.7 | 5.8 | 6.9 | 7.6 | 5.6 | 7.9 | ***6.7*** | *7.0* | *0.9* |
| mean | ***2.7*** | ***2.8*** | ***1.0*** | ***2.5*** | ***2.5*** | ***2.2*** | ***1.9*** | ***2.2*** | ***2.3*** | ***1.9*** | ***1.4*** | ***2.5*** | 2.1 | | |
| median | *3.4* | *3.8* | *2.0* | *3.2* | *3.4* | *2.9* | *2.4* | *2.9* | *3.0* | *2.0* | *1.9* | *3.2* | | 3.0 | |
| sd | *3.8* | *3.6* | *3.8* | *3.2* | *3.4* | *3.2* | *3.0* | *3.3* | *3.7* | *3.8* | *3.3* | *4.0* | | | 0.4 |
| *month stats* | | | | | | | | | | | | | | | |
