# Supplementary material for: Variation in ultraviolet radiation and diabetes: evidence of an epigenetic effect that modulates diabetics’ lifespan
Source: Clin Epigenetics. 2013 Apr 2;5(1):5. doi: 10.1186/1868-7083-5-5 (PMC3639074; doi:10.1186/1868-7083-5-5)
Supplement: Additional file 2: Appendix 1. Table S1 — [MAX – MIN] for White Non-diabetics & Diabetics by Increasing or Decreasing light at Birth (or Conception). Table S2: [MAX – MIN] for Asian Non-diabetics & Diabetics by Increasing or Decreasing light at Birth (or Conception). Table S3: [MAX – MIN] for White (Canadian) Non-diabetics & Diabetics by Increasing or Decreasing light at Birth (or Conception). Table S4: [MAX – MIN] for Hispanic Non-diabetics & Diabetics by Increasing or Decreasing light at Birth (or Conception). Table S5: [MAX – MIN] for African-American Non-diabetics & Diabetics by Increasing or Decreasing light at Birth (or Conception). Table S6: [MAX – MIN] for Native American Non-diabetics & Diabetics by Increasing or Decreasing light at Birth (or Conception). [file 1868-7083-5-5-S2.pdf]

APPENDIX 1: **TABLE 1: [MAX – MIN] for White Non-diabetics & Diabetics by Increasing or Decreasing light at Birth (or Conception)**

**WHITE**	Average MAX-MIN JUL-AUG BIRTHS (YRS)	Average MAX-MIN NOV-DEC BIRTHS (YRS)
ALL MALE (WHITE) NON-Diabetic	**-3.167** Avg. age MAX: 67.4 Avg. age at MIN: 70.6 N max = 815,000 N min = 795,000 Std err = 0.03 P<0.0001	**-3.141** Avg. age MAX: 67.8 Avg. age at MIN: 71.0 N max = 756,000 N min = 726,000 Std err = 0.03 P<0.0001
ALL FEMALE NON-Diabetic	**-2.479** Avg. age MAX: 74.7 Avg. age at MIN: 77.2 N max = 759,000 N min = 796,000 Std err = 0.03 P<0.0001	**-2.360** Avg. age MAX: 75.2 Avg. age at MIN: 77.5 N max = 708,000 N min = 730,000 Std err = 0.03 P<0.0001
DIABETIC MALE	**-2.610** Avg. age MAX: 68.7 Avg. age at MIN: 71.3 N max = 9,756 N min = 9,783 Std err = 0.19 P<0.0001	**-2.057** Avg. age MAX: 69.6 Avg. age at MIN: 71.6 N max = 9,117 N min = 9,150 Std err = 0.20 P<0.0001
DIABETIC FEMALE	**-2.074** Avg. age MAX: 73.6 Avg. age at MIN: 75.4 N max = 11,493 N min = 12,391 Std err = 0.16 P<0.0001	**-1.789** Avg. age MAX: 74.0 Avg. age at MIN: 75.8 N max = 11,053 N min = 11,936 Std err = 0.17 P<0.0001
Equivalent months of conception	OCT-NOV (decreasing light)	FEB-MAR (increasing light)

APPENDIX 1 TABLE 2: [MAX – MIN] for Asian Non-diabetics & Diabetics by Increasing or Decreasing light at Birth (or Conception)

ASIAN	Average MAX-MIN JUL-AUG BIRTHS (YRS)	Average MAX-MIN NOV-DEC BIRTHS (YRS)
ALL MALE NON-Diabetic	**-5.211**<br>Avg. age MAX: 61.7<br>Avg. age at MIN: 66.9<br>N max = 17,901<br>N min = 40,857<br>Std err = 0.20<br>Pr > |t| <0.0001	**-5.444**<br>Avg. age MAX: 62.0<br>Avg. age at MIN: 67.5<br>N max = 18,191<br>N min = 42,344<br>Std err = 0,.20<br>Pr > |t| <0.0001
ALL FEMALE NON-Diabetic	**-4.628**<br>Avg. age MAX: 65.4<br>Avg. age at MIN: 70.0<br>N max = 13,466<br>N min = 32,100<br>Std err = 0.23<br>Pr > |t| <0.0001	**-4.537**<br>Avg. age MAX: 66.2<br>Avg. age at MIN: 70.7<br>N max = 14,180<br>N min = 33,992<br>Std err = 0.22<br>Pr > |t| <0.0001
DIABETIC MALE	**-1.889**<br>Avg. age MAX: 70.1<br>Avg. age at MIN: 72.0<br>N max = 205<br>N min = 565<br>Std err = 1.03<br>Pr > |t| 0.067	**-3.630**<br>Avg. age MAX: 68.8<br>Avg. age at MIN: 72.4<br>N max = 218<br>N min = 549<br>Std err = 0.98<br>Pr > |t| <0.0002
DIABETIC FEMALE	**-3.691**<br>Avg. age MAX: 69.9<br>Avg. age at MIN: 73.6<br>N max = 234<br>N min = 578<br>Std err = 0.96<br>Pr > |t| <0.0001	**-1.842**<br>Avg. age MAX: 71.8<br>Avg. age at MIN: 73.6<br>N max = 226<br>N min = 624<br>Std err = 0.99<br>Pr > |t| 0.062
Equivalent months of conception	OCT-NOV (decreasing light)	FEB-MAR (increasing light)

APPENDIX 1 TABLE 3: [MAX – MIN] for White (Canadian) Non-diabetics & Diabetics by Increasing or Decreasing light at Birth (or Conception)

**CANADIAN WHITE**	Average MAX-MIN JUL-AUG BIRTHS (YRS)	Average MAX-MIN NOV-DEC BIRTHS (YRS)
ALL MALE NON-Diabetic	**-2.523**<br>Avg. age MAX: 74.1<br>Avg. age at MIN: 76.6<br>N max = 9,175<br>N min = 25,344<br>*std err = 0.16*<br>P<0.0001	**-2.749**<br>Avg. age MAX: 74.1<br>Avg. age at MIN: 76.9<br>N max = 8,140<br>N min = 21,982<br>*std err = 0.17*<br>P<0.0001
ALL FEMALE NON-Diabetic	**-1.418**<br>Avg. age MAX: 80.3<br>Avg. age at MIN: 81.7<br>N max = 11,509<br>N min = 32,882<br>*std err = 0.13*<br>Pr > |t|<0.0001	**-1.614**<br>Avg. age MAX: 80.4<br>Avg. age at MIN: 82.1<br>N max = 10,440<br>N min = 29,375<br>*std err = 0.14*<br>Pr > |t| <0.0001
DIABETIC MALE	**-2.935**<br>Avg. age MAX: 73.5<br>Avg. age at MIN: 76.4<br>N max = 126<br>N min = 291<br>*std err = 1.20*<br>Pr> |t| 0.0148	**-2.064**<br>Avg. age MAX: 75.0<br>Avg. age at MIN: 77.0<br>N max = 125<br>N min = 250<br>*std err = 1.04*<br>Pr > |t| 0.0473
DIABETIC FEMALE	**-0.643**<br>Avg. age MAX: 78.1<br>Avg. age at MIN: 78.8<br>N max = 144<br>N min = 408<br>*std err = 1.10*<br>Pr > |t| 0.558	**-2.016**<br>Avg. age MAX: 78.6<br>Avg. age at MIN: 80.6<br>N max = 161<br>N min = 385<br>*std err =0.98*<br>Pr > |t| 0.040
Equivalent months of conception	OCT-NOV (decreasing light)	FEB-MAR (increasing light)

APPENDIX 1 TABLE 4: [MAX – MIN] for Hispanic Non-diabetics & Diabetics by Increasing or Decreasing light at Birth (or Conception)

HISPANIC	Average MAX-MIN JUL-AUG BIRTHS (YRS)	Average MAX-MIN NOV-DEC BIRTHS (YRS)
ALL MALE NON-Diabetic	**-6.43**<br>Avg. age MAX: 54.8<br>Avg. age at MIN: 61.2<br>N max = 15,878<br>N min = 33,638<br>*Std err = 0.23*<br>P<0.0001	**-6.60**<br>Avg. age MAX: 55.5<br>Avg. age at MIN: 62.1<br>N max = 15,222<br>N min = 31,181<br>*Std err = 0.23*<br>P<0.0001
ALL FEMALE NON-Diabetic	**-5.07**<br>Avg. age MAX: 69.0<br>Avg. age at MIN: 74.0<br>N max = 10,142<br>N min = 26,392<br>*Std err = 0.23*<br>P<0.0001	**-4.88**<br>Avg. age MAX: 69.5<br>Avg. age at MIN: 74.4<br>N max = 10,159<br>N min = 24,468<br>*Std err = 0.23*<br>P<0.0001
DIABETIC MALE	**-3.15**<br>Avg. age MAX: 68.7<br>Avg. age at MIN: 71.9<br>N max = 239<br>N min = 663<br>*Std err = 0.95*<br>Pr> |t| 0.001	**-2.40**<br>Avg. age MAX: 69.4<br>Avg. age at MIN: 70.8<br>N max = 241<br>N min = 565<br>*Std err = 1.00*<br>Pr> |t| 0.017
DIABETIC FEMALE	**-1.27**<br>Avg. age MAX: 73.6<br>Avg. age at MIN: 74.8<br>N max = 320<br>N min = 889<br>*Std err = 0.80*<br>Pr> |t| 0.113	**-1.80**<br>Avg. age MAX: 73.8<br>Avg. age at MIN: 75.6<br>N max = 339<br>N min = 857<br>*Std err = 0.79*<br>Pr> |t| 0.022
Equivalent months of conception	OCT-NOV (decreasing light)	FEB-MAR (increasing light)

APPENDIX1 TABLE 5: [MAX – MIN] for African-American Non-diabetics & Diabetics by Increasing or Decreasing light at Birth (or Conception)

**AFRICAN-AMERICAN**	Average MAX-MIN JUL-AUG BIRTHS (YRS)	Average MAX-MIN NOV-DEC BIRTHS (YRS)
ALL MALE NON-Diabetic	**-5.609**<br>Avg. age MAX: 55.5<br>Avg. age at MIN: 61.1<br>N max = 189,000<br>N min = 402,000<br>Std err = 0.06<br>P<0.0001	**-5.180**<br>Avg. age MAX: 56.1<br>Avg. age at MIN: 61.2<br>N max = 175,000<br>N min = 366,000<br>Std err = 0.07<br>P<0.0001
ALL FEMALE NON-Diabetic	**-5.496**<br>Avg. age MAX: 62.9<br>Avg. age at MIN: 68.4<br>N max = 159,000<br>N min = 370,000<br>Std err = 0.07<br>P<0.0001	**-5.353**<br>Avg. age MAX: 63.4<br>Avg. age at MIN: 68.7<br>N max = 147,000<br>N min = 338,000<br>Std err = 0.07<br>P<0.0001
DIABETIC MALE	**-3.598**<br>Avg. age MAX: 63.4<br>Avg. age at MIN: 67.0<br>N max = 2,752<br>N min = 6,430<br>Std err = 0.32<br>P<0.0001	**-3.651**<br>Avg. age MAX: 63.6<br>Avg. age at MIN: 67.3<br>N max = 2,597<br>N min = 6,130<br>Std err = 0.33<br>P<0.0001
DIABETIC FEMALE	**-2.887**<br>Avg. age MAX: 68.5<br>Avg. age at MIN: 71.4<br>N max = 4,237<br>N min = 10,986<br>Std err = 0.24<br>P<0.0001	**-2.929**<br>Avg. age MAX: 68.6<br>Avg. age at MIN: 71.5<br>N max = 4,164<br>N min = 10,266<br>Std err = 0.25<br>P<0.0001
Equivalent months of conception	OCT-NOV (decreasing light)	FEB-MAR (increasing light)

APPENDIX 1 TABLE 6: [MAX – MIN] for Native American Non-diabetics & Diabetics by Increasing or Decreasing light at Birth (or Conception)

**NATIVE AMERICAN**	Average MAX-MIN JUL-AUG BIRTHS (YRS)	Average MAX-MIN NOV-DEC BIRTHS (YRS)
ALL MALE NON-Diabetic	**-5.366**<br>Avg. age MAX: 51.8<br>Avg. age at MIN: 57.2<br>N max = 7,632<br>N min = 15,309<br>Std err = 0.34<br>P <0.0001	**-5.796**<br>Avg. age MAX: 51.2<br>Avg. age at MIN: 57.0<br>N max =6,946<br>N min = 13,786<br>Std err = 0.36<br>P <0.0001
ALL FEMALE NON-Diabetic	**-6.307**<br>Avg. age MAX: 58.3<br>Avg. age at MIN: 64.6<br>N max = 5,410<br>N min = 12,464<br>Std err = 0.40<br>P <0.0001	**-5.039**<br>Avg. age MAX: 58.8<br>Avg. age at MIN: 63.9<br>N max = 5,030<br>N min = 11,313<br>Std err = 0.42<br>P <0.0001
DIABETIC MALE	**-3.455**<br>Avg. age MAX: 61.9<br>Avg. age at MIN: 65.3<br>N max = 127<br>N min = 377<br>Std err = 1.41<br>Pr > |t| 0.014	**-2.294**<br>Avg. age MAX: 63.3<br>Avg. age at MIN: 65.6<br>N max = 106<br>N min = 317<br>Std err = 1.47<br>Pr > |t| 0.120
DIABETIC FEMALE	**-2.926**<br>Avg. age MAX: 67.3<br>Avg. age at MIN: 70.2<br>N max = 207<br>N min = 481<br>Std err = 1.06<br>Pr > |t| 0.006	**-1.507**<br>Avg. age MAX: 68.0<br>Avg. age at MIN: 69.5<br>N max = 195<br>N min = 447<br>Std err = 1.05<br>Pr > |t| 0.151
Equivalent months of conception	OCT-NOV (decreasing light)	FEB-MAR (increasing light)
